# Toosendanin From Melia Fructus Suppresses Influenza A Virus Infection by Altering Nuclear Localization of Viral Polymerase PA Protein

**DOI:** 10.3389/fphar.2019.01025

**Published:** 2019-09-12

**Authors:** Young-Hee Jin, Sunoh Kwon, Jang-Gi Choi, Won-Kyung Cho, Bonggi Lee, Jin Yeul Ma

**Affiliations:** ^1^KM Application Center, Korea Institute of Oriental Medicine (KIOM), Daegu, South Korea; ^2^Herbal Medicine Research Division, Korea Institute of Oriental Medicine (KIOM), Daejeon, South Korea; ^3^Center for Convergent Research of Emerging Virus Infection (CEVI), Korea Research Institute of Chemical Technology (KRICT), Daejeon, Republic of Korea

**Keywords:** toosendanin, Melia Fructus, influenza A virus, polymerase acidic protein, anti-viral activity

## Abstract

Toosendanin (TSN) is a major bioactive component of Melia Fructus (MF) with anti-inflammatory, anti-botulinum, anti-microbial, and analgesic efficacy. Our previous study demonstrated that MF has anti-influenza A virus activity; however, the contribution of TSN is still unclear. In this study, we found that TSN suppressed influenza A virus infection when administered before or concurrent with the virus, but not after infection. TSN pretreatment inhibited viral hemagglutinin (HA), nucleoprotein (NP), polymerase acidic (PA) protein, and matrix protein 2 (M2) mRNA synthesis as well as NP, PA, M2, and nonstructural protein 1 (NS1) expression but had no effect on HA or neuraminidase (NA) activity. In addition, TSN induced cytoplasmic location of PA protein disrupting nuclear translocation. Docking simulation suggested that the binding affinity of TSN to PA protein may be stronger than that of a known PA protein inhibitor. Pretreatment with TSN also suppressed the infection-induced phospho-AKT expression but not the host immune response. Oral pretreatment with TSN enhanced the survival of infected mice. These results suggest that TSN inhibits influenza A virus infection at an early stage by altering PA protein nuclear localization. Thus, TSN may be a promising candidate for anti-influenza agent targeting the PA protein of the influenza A virus RNA polymerase complex.

## Introduction

Influenza A virus, a single-stranded RNA virus belonging to the family *Orthomyxoviridae*, causes seasonal and pandemic morbidity and mortality. Drugs targeting viral protein for the treatment of influenza are available, and others are in development. The viral matrix protein 2 (M2) has low pH-activated H^+^ channel activity and is involved in uncoating of the virion (Helenius, 1992). The first available drugs for influenza treatment were M2 ion channel inhibitors, rimantadine, and amantadine, but these are no longer recommended because of side effects and emergence of resistance (von Itzstein, 2007). The hemagglutinin (HA) protein mediates binding of virus to host receptor sialyloligosaccharide, and the neuraminidase (NA) protein facilitates viral particle release by cleaving sialyloligosaccharide (Paules and Subbarao, 2017). The HA inhibitor, *tert*-butylhydroquinone inhibits the conformational rearrangements required for membrane fusion, but it binds in a hydrophobic pocket of H3HA specifically, not H1HA or H5HA (Russell et al., 2008). NA inhibitors, zanamivir, oseltamivir, laninamivir, and peramivir, are currently available although emergence of resistance stains by mutation, and reassortment has been reported (Thorlund et al., 2011).

The influenza A genome consists of eight RNA segments, and each segment is packed in a ribonucleoprotein (RNP) complex with viral nucleoprotein (NP) and the three RNA-dependent RNA polymerase subunits, polymerase acidic (PA) protein, and the two polymerase basic (PB1 and PB2) proteins. PB1 possesses RNA polymerization activity, PB2 recognizes and binds the host 5′ mRNA cap structure, and PA has endonucleolytic activity and can induce proteolysis (Rodriguez-Frandsen et al., 2015). Replication and transcription (−vRNA → +cRNA and –vRNA → mRNA) occur in the nucleus of virus-infected cells, and the absence of a viral polymerase proofreading function frequently creates mutations (Krug and Aramini, 2009). Influenza virus polymerase subunits are an attractive target for anti-influenza viral agents, because polymerase subunit is conserved in influenza virus strains and plays an important role on replication and transcription of viral genome. Polymerase inhibitors would block viral replication and transcription during the early stage of infection. For instance, favipiravir (T-705) blocks the RNA synthesis activity of PB1 (Furuta et al., 2013) and a substituted 2,6-diketopiperazine natural compound (flutamide) and the latest FDA-approved antiviral drug, baloxavir marboxil (Xofluza), were shown to inhibit endonuclease activity of PA, preventing the transcription of viral mRNA (Tomassini et al., 1996; O’Hanlon and Shaw, 2019). Although vaccines are available for seasonal influenza prevention, they do not give adequate protection, due to antigenic mismatch and low effectiveness (Lewnard and Cobey, 2018). Therefore, the development of new antiviral drugs targeting polymerase subunit is essential for treatment and prophylaxis without viral resistance.

Our previous study demonstrated that Melia Fructus (MF) has anti-influenza A virus activity by blocking viral entry and altering the expression and subcellular localization of RNA polymerase proteins (Jin et al., 2017). MF is used as a traditional medicine to treat stomachache and acute or chronic inflammation, and as an anthelmintic and antifeedant (Xie et al., 2008). Toosendanin (TSN) is an active limonoid ingredient from MF with numerous bioactivities (Tada et al., 1999; Shi and Li, 2007). For instance, MF and two limonoids were reported to have anti-inflammatory and analgesic activities (Xie et al., 2008). In addition, TSN suppressed proliferation and induced apoptosis of human cancer cells (Zhang et al., 2005) and displayed anti-botulinum and anti-microbial activities as well as antifeedant and insecticide properties (Carpinella et al., 2003; Shi and Wang, 2004; Kamau et al., 2016). TSN also inhibited hepatitis C virus infection by enhancing alpha interferon (Watanabe et al., 2011). Another MF compound, 28-deacetylsendanin, inhibited the replication of herpes simplex virus-1 (Kim et al., 1999). Limonoids from MF also showed potent antiviral activity against West Nile virus, Dengue virus, and yellow fever virus as well as antimycobacterial activity against *Mycobacterium tuberculosis* (Sanna et al., 2015). However, the effect of TSN on influenza A virus infection is still unclear. In this study, we examined the effect of TSN on influenza A virus infection, the underlying mechanisms of action, and the efficacy of TSN against infection in a rodent model. We suggest that TSN is a promising candidate for anti-influenza agent targeting the PA protein of the influenza A virus RNA polymerase complex.

## Materials and Methods

### Ultra-Performance Liquid Chromatography/Mass Spectrometry Analysis

The ethanol extract of MF was prepared as described previously (Jin et al., 2017). TSN and Ohchinin (OCN) were purchased from ChemFaces (Wuhan, China) (purity >98%) and used as experimental compounds and standards for ultra-performance liquid chromatography/mass spectrometry (UPLC/MS) (Akihisa et al., 2013). The components of MF were separated using by a several Waters ACQUITY UPLC H-Class modules (Waters Corporation, Milford, MA, USA) coupled to the SQ detector 2 mass spectrometer (Waters Corporation, Milford, MA, USA) with electrospray ionization (ESI). Chromatographic separation was achieved with a Waters Acquity UPLC BEH C_18_ octadecylsilane column (2.1 mm × 100 mm, 1.7 µm) and mobile phase (80% B) using 10 mM ammonium acetate in water or acetonitrile. The MS conditions were as follows: ion spray voltage, 3.5 kV; capillary voltage, 20 V; capillary temperature, 350°C; and tube lens voltage, 40 V. Analysis for TSN and OCN was performed in negative and positive mode, respectively (Ong and Ong, 2007).

### Cells and Viruses

Madin–Darby canine kidney (MDCK) cells (American Type Culture Collection, ATCC CCL-34; Manassas, VA) were cultured in Eagle’s Minimum Essential medium (Lonza, Allendale, NJ) containing 10% fetal bovine serum (FBS), 100 U/ml penicillin, and 100 µg/ml streptomycin at 37°C in 5% CO_2_ incubator. The influenza A viruses Puerto Rico/8/34 (A/PR/8/34; H1N1), KBPV-VR-32 (H3N2), green fluorescent protein (GFP)-tagged A/PR/8/34 (A/PR/8/34-GFP) (Jin et al., 2017), and mouse-adapted challenge virus (A/PR/8/34) were amplified in the allantoic fluid of 10-day-old chicken embryos. A/PR/8/34, A/PR/8/34-GFP, and mouse-adapted challenge viruses were kindly provided by Professor Jong-Soo Lee (Chungnam National University, Daejeon, Korea). The KBPV-VR-32 (H3N2) strain was obtained from the Korea Bank for pathogenic viruses.

### MTS Cytotoxicity Assay

MDCK cells were seeded in 96-well culture plates at 2 × 10^4^ cells/well and incubated at 37°C overnight with 5% CO_2_. TSN or OCN was added at the indicated concentrations for 24 h (up to 10 microM). A 3-(4,5-dimethylthiazol-2-yl)-5-(3-carboxymethoxyphenyl)-2-(4-sulfophenyl)-2*H*-tetrazolium (MTS) solution (Promega, Madison, WI) was added for 1 h. Absorbance was measured at 490 nm by a GloMax Explorer System (Promega, Madison, WI).

### Influenza A Virus Infection

In pretreatment assays, MDCK cells were treated with TSN or OCN at the indicated concentrations for 6 h, washed with phosphate-buffered saline (PBS), and infected with 10 multiplicity of infection (MOI) virus for 2 h at 37°C. For cotreatment assays, the compounds were mixed with 10 MOI influenza A virus, and the mixture was incubated at 4°C for 1 h. Then, MDCK cells were inoculated with the virus mixture at 37°C for 2 h. In posttreatment assays, the cells were first infected with 10 MOI viruses for 2 h at 37°C. The virus was then washed away with PBS, and cells were treated with the indicated concentrations of TSN or OCN. After all treatments, MDCK cells were incubated in culture media containing l-1-tosylamide-2-phenylethyl chloromethyl ketone (TPCK)–treated trypsin (Sigma, St. Louis, MO) at 10 µg/ml for the indicated time at 37°C under 5% CO_2_ (**Figure 2A**).

### Antiviral Assay

Viral cytopathic effect (CPE) was examined at 24-h postinfection using the MTS assay as described above. After A/PR/8/34-GFP virus infection, viral GFP expression was analyzed by a Nikon ECLIPSE Ti fluorescence microscope (Nikon, Tokyo, Japan), and GFP intensity was determined using a GloMax Explorer System (Promega, Madison, WI). We calculated 50% effective concentrations (EC_50_) values for TSN and OCN by regression analysis.

### Hemagglutination Inhibition Assay

Influenza A virus A/PR/8/34 or H3N2 was diluted at 4 HAU/25 µl and mixed with 25 µl serially diluted TSN in 96-well round bottom plates for 1 h at 4°C. Then, 50 µl of 0.5% chicken red blood cells (cRBCs) (Innovative Research, Southfield, MI) diluted in PBS was added to each well. Reaction mixtures were incubated at 25°C for 1 h and then photographed.

### Neuraminidase Inhibition Assay

NA inhibition assays were conducted using the NA-Fluor™ Influenza Neuraminidase Assay Kit (Applied Biosystems, Foster City, CA) according to the manufacturer’s instructions. Serially diluted TSN in assay buffer was added to black 96-well plates at 25 µl/well. Then, 25 µl A/PR/8/34 or H3N2 was added to each well, and the mixture was incubated for 30 min at 37°C, followed by 50 µl of 200 µM NA-Fluor Substrate. After 1 h incubation at 37°C, 100 µl NA-Fluor stop solution was added to terminate the reaction and was monitored by GloMax Explorer System (Promega, Madison, WI).

### Quantitative Real-Time Polymerase Chain Reaction

Total RNA was extracted using the RNeasy Mini Kit (Qiagen, Hilden, Germany) and samples (500 ng) mixed with RT-PreMix (Bioneer, Daejeon, Korea) and OligodT_18_ (Bioneer, Daejeon, Korea) to synthesize cDNA. Quantitative real-time polymerase chain reaction (qRT-PCR) was conducted using the Touch Real-Time PCR System (Bio-Rad, Hercules, CA) with qPCR Master Mix (Bioneer, Daejeon, Korea) and the following primers: HA (5′-ttgctaaaacccggagacac-3′ and 5′-cctgacgtatttgggcact-3′); NP (5′-gaatggtgctctctgcttttga-3′ and 5′-tccactttccgtttactctcctg-3′); PA (5′-aagtgccataggccaggtttc-3′ and 5′-cctcatctccattccccatttc-3′); M2 (5′-gaaaggagggccttctacgg-3′ and 5′-tcgtcagcatccacagcac-3′); canine IFN-β (5′-ccagttccagaaggaggaca-3′ and 5′-tgtcccaggtgaagttttcc-3′); canine Mx1 (5′-gaatcctgtacccaatcatgtg-3′ and 5′-taccttctcctcatattggct-3′); and canine β-actin (5′-tgccttgaagttggaaaacg-3′ and 5′-ctggggcctaatgttctcaca-3′) (Shoji et al., 2015). β-Actin expression was used as an internal reference, and relative expression of each gene was calculated using the ΔΔCt method. Every reaction was performed in triplicate.

### Immunofluorescence Staining

MDCK cells were fixed in 10% formalin solution for 10 min and permeabilized in 0.3% Triton X-100 for 15 min. After blocking the cells with 5% bovine serum albumin, cells were then incubated with anti-M2, -PA, -NS1, -NP, PB1, and PB2 (all from GeneTex, San Antonio, TX), or anti-phospho-AKT (Ser473) antibody (Cell Signaling Technology, Danvers, MA) overnight at 4°C, and then incubated with an Alexa Fluor^®^ 488 tagged anti-rabbit IgG antibody (Thermo Scientific, Danvers, MA) at 37°C for 1 h. Nuclei were counterstained using 1 µg/ml DAPI. Images were analyzed using a Nikon ECLIPSE Ti Fluorescence Microscope (Nikon, Tokyo, Japan) and a NIS-Elements Imaging Software (Nikon, Tokyo, Japan).

### *In Silico* Analysis of Compounds

The AutoDock Vina program was used for *in silico* protein–ligand docking simulation. The 3D structure of the 2009 H1N1 pandemic strain (pH1N1) virus PA N-terminal (PA-Nter) domain was obtained from the Protein Data Bank (accession no. 4WAK). The predefined binding site of PA-Nter domain was used as a docking pocket (Kowalinski et al., 2012). To predict the relative binding affinity of TSN with PA-Nter domain, we used 4-[3-[(4-chlorophenyl)methyl]-1-(phenylmethylsulpho)-3-piperidinyl]-2-hydroxy-4-oxo-2-butenoic acid (R05-01), a known diketo inhibitor that chelates the two manganese ions in the active site of PA protein, as a positive control (Kowalinski et al., 2012). After the protein–ligand docking simulation, generation of pharmacophores was analyzed using LigandScout 3.0 software.

### Oral Administration of TSN and Virus Infection in BALB/c Mice

Female 5-week-old BALB/c mice were purchased from Orient Bio (Gyeonggi, Korea) and acclimated for 7 days before experiments. TSN were prepared in 0.5% sodium carboxymethyl cellulose (CMC) and orally administered to the mice (1 mg/kg) in a total volume of 200 µl at −7 to 0 day before infection (*n* = 10 mice at each dose). Vehicle control (0.5% CMC, 200 µl) was orally administered to control group mice (*n* = 10). Drug treatment and infection were performed in an approved ABSL-2+ laboratory facility. Mice were infected intranasally with 20 µl A/PR/8/34 at five times the 50% minimum lethal dose (MLD_50_). Body weight and survival were monitored for 17 days postinfection. All animal protocols were approved by the Institutional Animal Care and Use Committee of the Daegu-Gyeongbuk Medical Innovation Foundation (DGMIF), Daegu, South Korea (approval number, DGMIF-17031401-01).

### Statistical Analysis

Means of more than two treatment groups were compared by one-way analysis of variance (ANOVA), whereas experimental and control group means were compared by unpaired Student’s *t*-test (two-tailed) using GraphPad Prism software version 5.0 (GraphPad Software, San Diego, CA). *P* < 0.05 was considered significant.

## Results

### Identification of Components in MF Ethanol Extract

**Figure 1:**
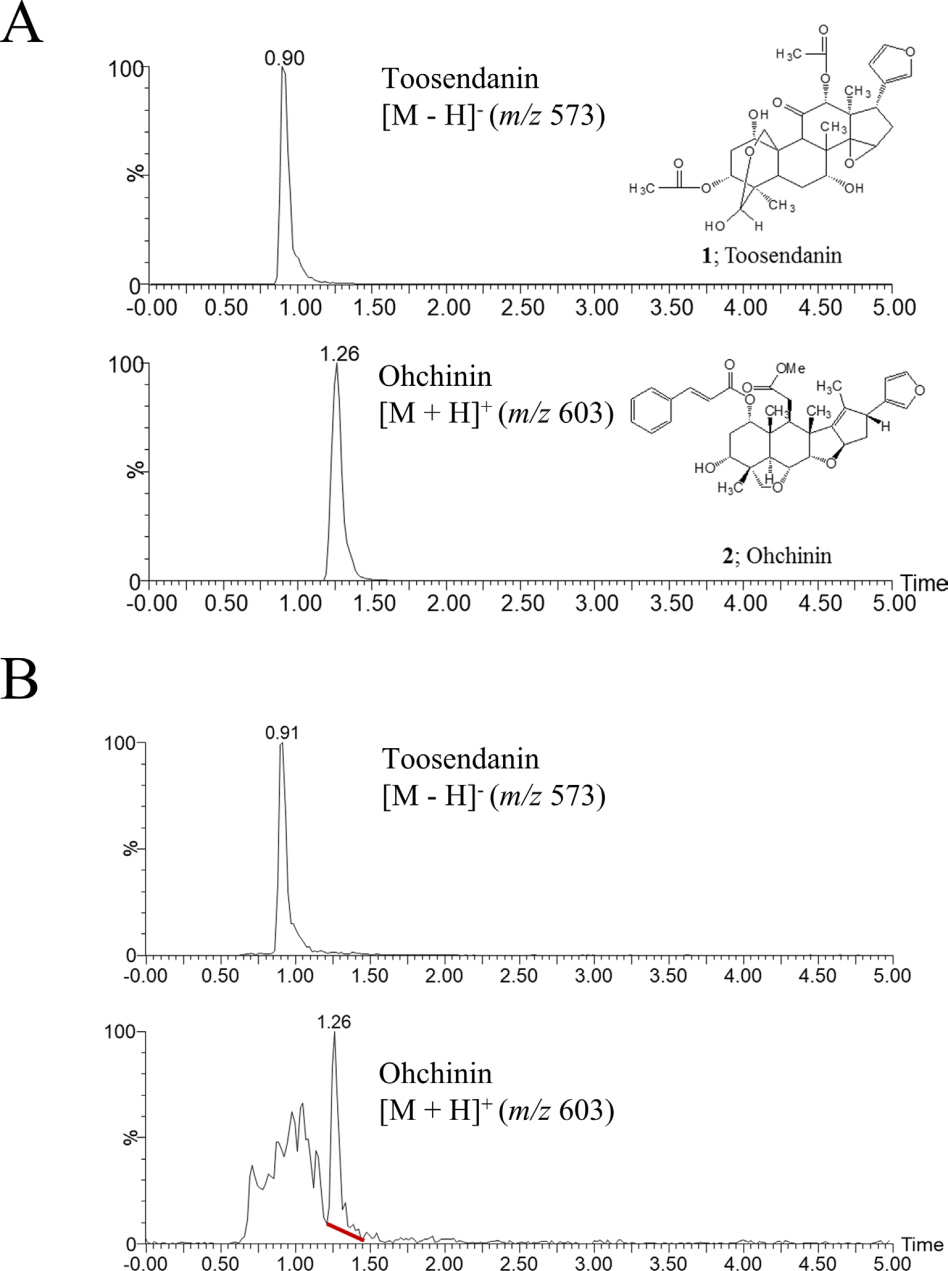
UPLC-MS chromatograms of toosendanin, Ohchinin, and Melia Fructus (MF) ethanol extract. **(A)** The chemical structures of toosendanin (TSN) and Ohchinin (OCN) and the UPLC-MS chromatograms of TNS (upper panel) and OCN (lower panel) standards in single ion monitoring (SIM) mode. **(B)** UPLC-MS chromatograms for m/z 573 (upper panel) and m/z 603 (lower panel) ions in MF ethanol extract.

We previously reported that the ethanol extract of MF has anti-influenza A virus activity (Jin et al., 2017). To identify the active component of MF, we performed UPLC-MS analysis. In UPLC-MS chromatograms in single ion monitoring (SIM) mode, the retention times (*t* *_R_*) of TSN and OCN standards were shown as 0.90 min at [M−H]^−^ (m/z 573) and 1.26 min at [M + H]^+^ (m/z 603), respectively (**Figure 1A**). Then, single peaks were identified in the ethanol extract of MF at retention times similar to the TSN and OCN standards (*t* *_R_* for TSN at 0.91 and OCN at 1.26) (**Figure 1B**). Therefore, we confirmed TSN and OCN as components of MF ethanol extract and subsequently examined whether these commercial compounds have host-cell toxicity and anti-influenza A virus activity.

### Cytotoxic Effects of TSN and OCN on MDCK Cells

**Figure 2:**
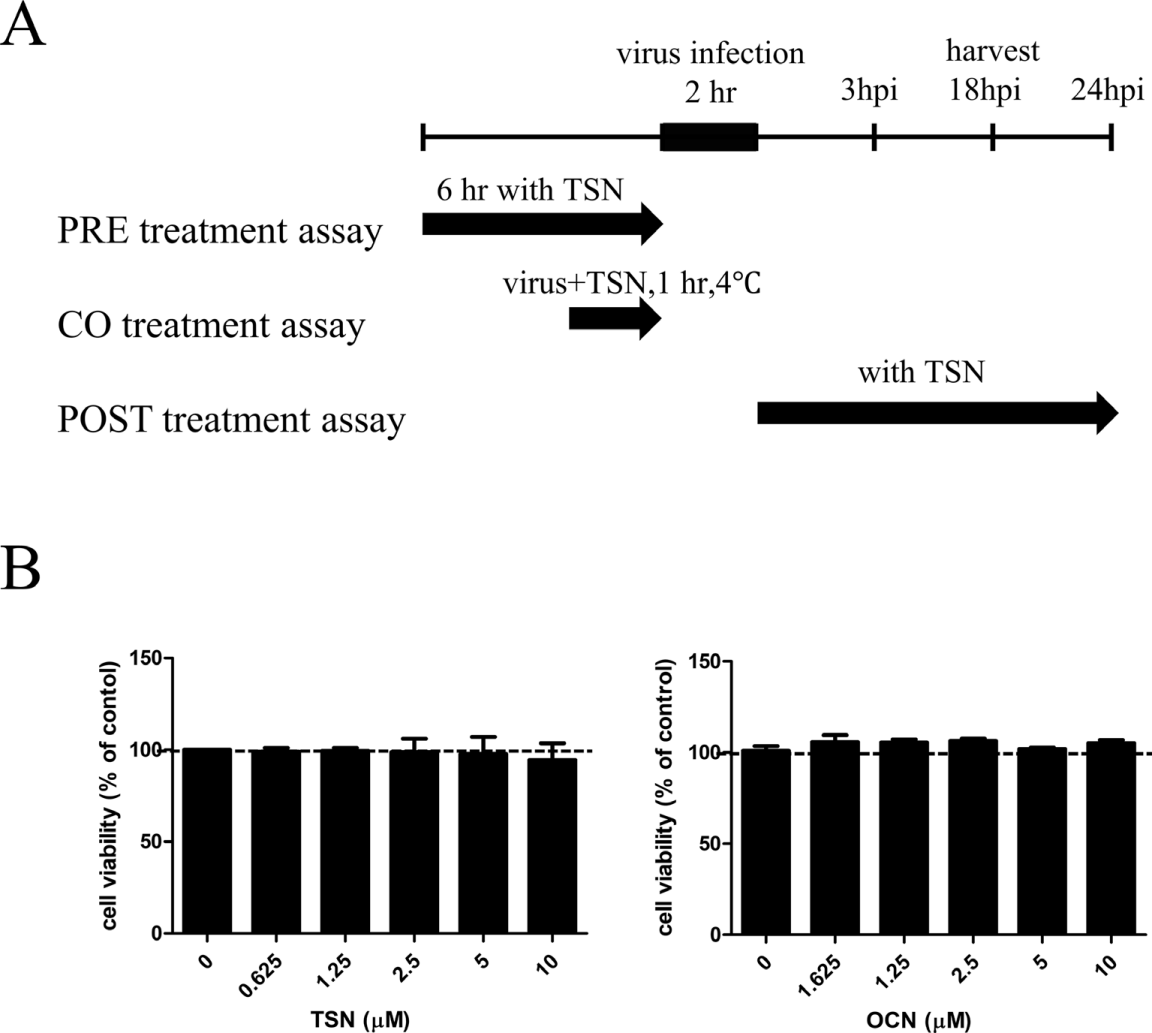
Schematic diagram of the anti-influenza virus assay and cytotoxicity assay for TSN and OCN in MDCK cells. **(A)** In the pretreatment assay, MDCK cells were treated with various TSN concentrations for 6 h before infection with 10 MOI influenza A virus. In the cotreatment assay, 10 MOI influenza virus and various concentrations of TSN were first mixed and incubated at 4°C for 1 h before addition to MDCK cell cultures for 2 h. In the posttreatment assay, MDCK cells were first infected with influenza A virus for 2 h at 37°C and then treated with TSN for the indicated time. **(B)** The viability of MDCK cells was analyzed by MTS assay after 24-h treatment with the indicated concentrations of TSN or OCN (up to 10 µM) (TSN CC_50_ > 100).

The cytotoxicity of various TSN and OCN concentrations on MDCK cells was analyzed by MTS assay after 24-h treatment. Neither TSN nor OCN exhibited cytotoxic effects on MDCK cells at concentrations up to 10 µM (**Figure 2B**). Thus, subsequent experiments on MDCK cells were performed at concentrations below 10 µM TSN or OCN.

### TSN But Not OCN Suppressed Influenza Virus Infection of MDCK Cells

**Figure 3:**
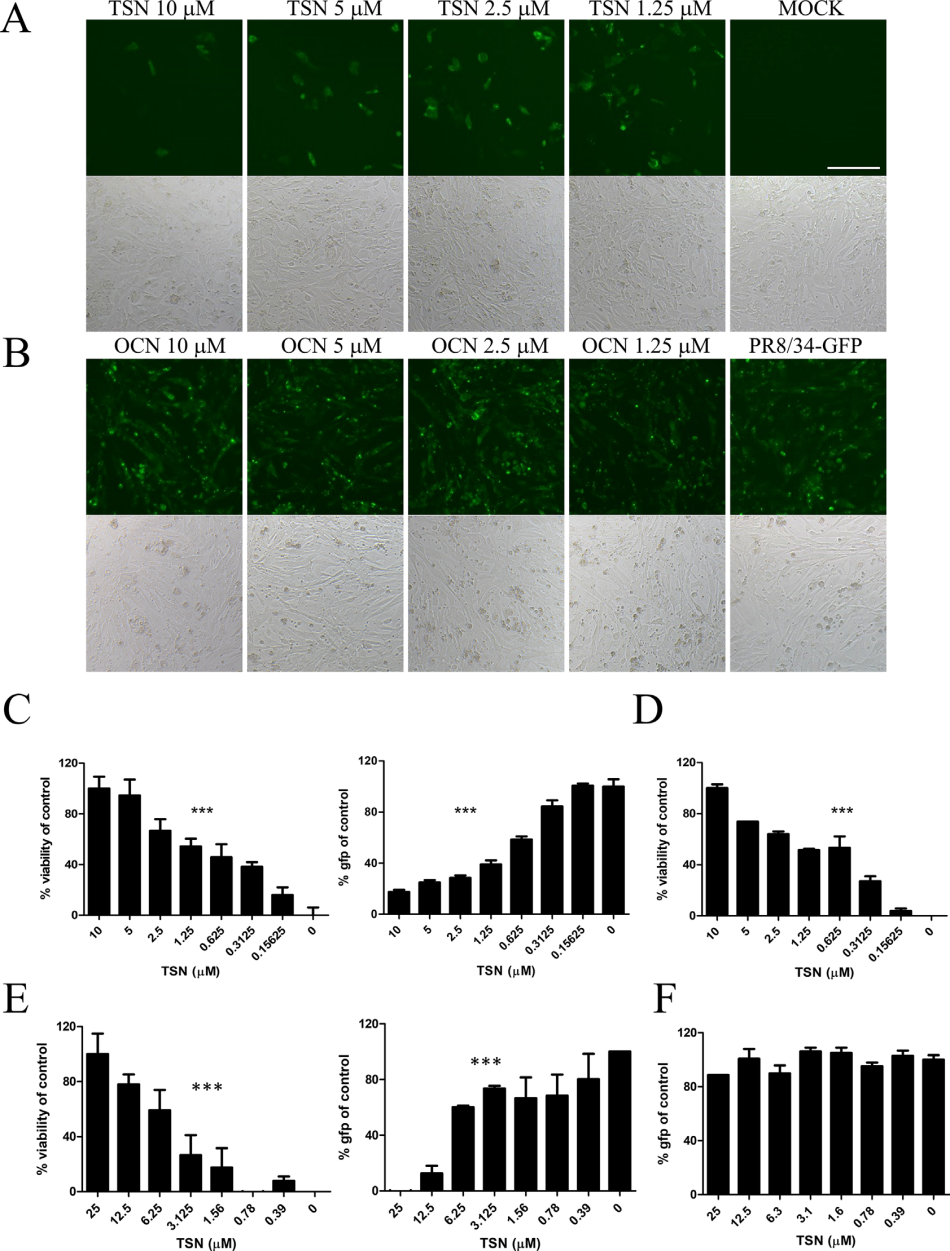
TSN suppressed influenza A virus infection in a dose-dependent manner. **(A** and **B)** In the pretreatment assay, MDCK cells were pretreated with various concentrations of TSN **(A)** or OCN **(B)** for 6 h prior to 10 MOI A/PR/8/34-GFP virus infection. The expression of viral GFP protein was detected by fluorescence microscopy (upper panel, fluorescence; bottom panel, phase contrast) at 24 h postinfection (hpi). Scale bars, 100 µm at ×20 magnification. **(C)** Measurement of the cytopathic effect (CPE) induced by 10 MOI A/PR/8/34-GFP virus in MDCKs cells pretreated with TSN using the MTS assay (left panel, IC_50_ = 0.76 microM) (non-virus-infected cell, 100% viability), and the expression of viral GFP was detected by fluorescence spectrometry (right panel, IC_50_ = 0.55 microM) at 24 hpi (GFP-virus-infected cell, 100% GFP value). **(D)** H3N2 (10 MOI) virus-induced CPE in TSN-pretreated MDCK cells was measured by MTS assay at 24 hpi. (IC_50_ = 0.48 microM) **(E)** In the cotreatment assay, MDCK cells were treated with a mixture of 10 MOI A/PR/8/34-GFP virus and TSN. CPE was measured by MTS assay (left panel, IC_50_ = 5.09 microM) and the expression of viral GFP protein by fluorescence spectrometry (right panel, IC_50_ = 3.34 mM) at 24 hpi. **(F)** In the posttreatment assay, MDCK cells were treated with TSN after 10 MOI A/PR/8/34-GFP infection, and CPE was measured by MTS assay at 24 hpi. The data are presented as mean ± SD of three replicates. All data are representative of three independent experiments. The statistical significance was analyzed by one-way ANOVA. ***P < 0.001.

Anti-influenza A virus activity was analyzed by monitoring the expression of viral GFP and by CPE assay of MDCK cells infected with A/PR8/34-GFP (H1N1) (Jin et al., 2017) and either pretreated, cotreated, or posttreated with TSN or OCN. In pretreatment experiments, MDCK cells were treated for 6 h with serially diluted concentrations (up to 10 µM) of TSN or OCN and then infected with 10 MOI A/PR8/34-GFP for 2 h. At 24-h postinfection (hpi), the expression of viral GFP was significantly reduced in a dose-dependent manner by TSN pretreatment (**Figures 3A, C**). Consistent with these results, the MTS cytotoxicity assay revealed that TSN pretreatment dose-dependently protected MDCK cells from virus-induced cell death (**Figure 3C**) as well as HEK 293T cells (**Supplementary Figure 2**). Regression analysis indicated that TSN pretreatment inhibited virus-induced GFP expression and CPE with an EC_50_ of 0.69 ± 0.1 µM at 24 hpi. Moreover, TSN also inhibited H3N2 influenza A virus-induced CPE in a dose-dependent manner with an EC_50_ of 0.63 ± 0.3 µM (**Figure 3D**). In the cotreatment assay, TSN still dose-dependently inhibited the expression of viral GFP and reduced cell death with an EC_50_ value of 3.6 ± 1.0 µM at 24 hpi (**Figure 3E**). Alternatively, TSN showed no inhibitory efficacy in the posttreatment assay condition (**Figure 3F**). OCN showed no inhibitory effect against influenza A virus even in the pretreatment assay (**Figure 3B**) as well as co- and posttreatment assays (data not shown). However, TSN treatment did not suppress other RNA virus, vesicular stomatitis virus (VSV), and Newcastle disease virus (NDV) virus infection (**Supplementary Figure 1**). Therefore, TSN pretreatment and cotreatment can dose-dependently inhibit influenza A virus infection specifically, whereas posttreatment is ineffective.

### TSN Did Not Inhibit Viral HA or NA Activity

**Figure 4:**
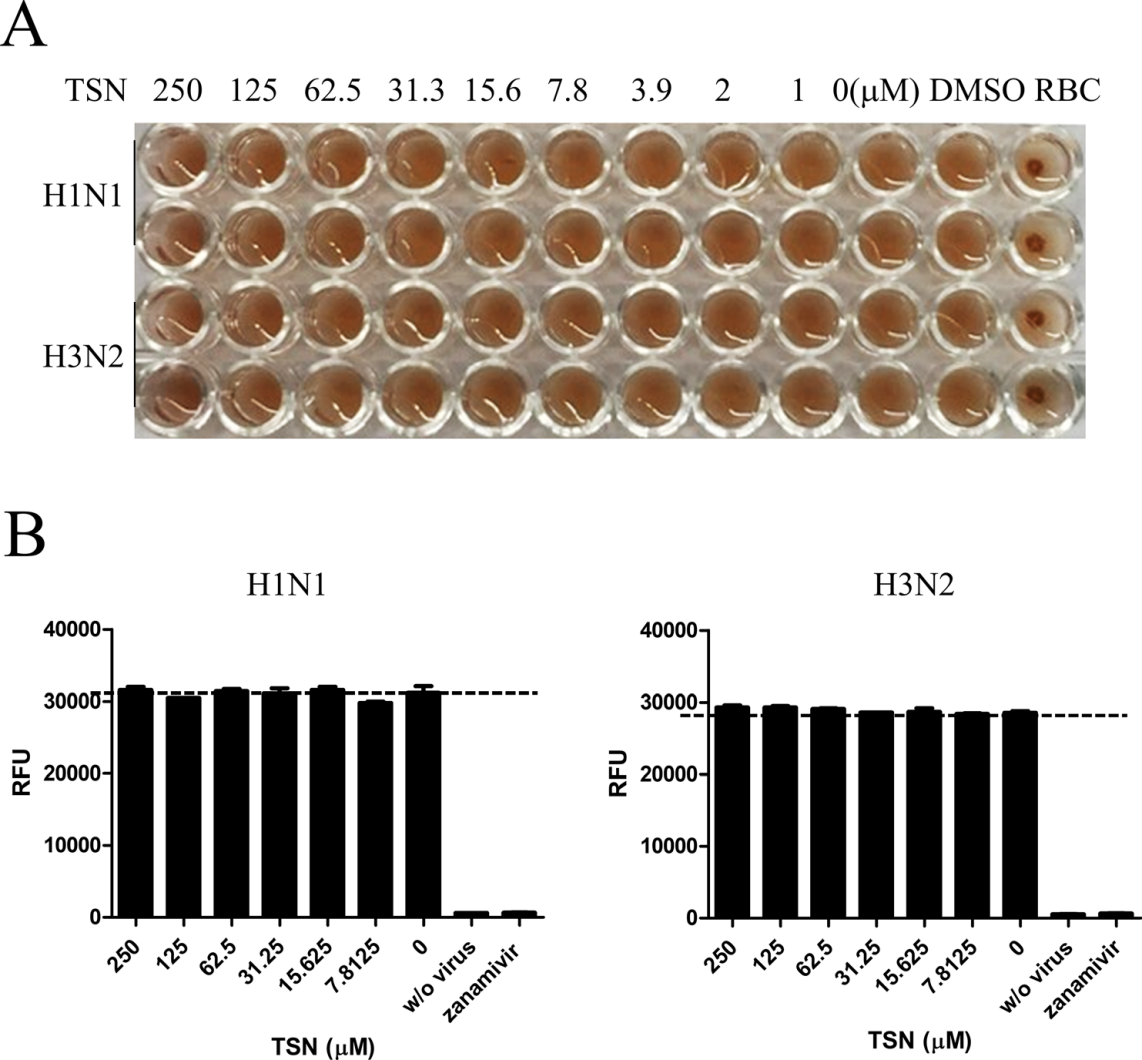
TSN did not inhibit the hemagglutinin (HA) or neuraminidase (NA) activity of influenza A virus strains A/PR/8/34 and H3N2. **(A)** Influenza virus A/PR/8/34 (upper two lanes) and H3N2 (lower two lanes) were diluted with PBS to 4 HAU and mixed with serially diluted concentrations (up to 250 µM) of TSN for 1 h at 4°C in duplicate. Chicken RBCs in PBS (0.5% w/v) were added and incubated for 1 h at room temperature. The plates were then photographed. **(B)** Influenza virus A/PR/8/34 or H3N2 in assay buffer was incubated with serially diluted TSN or the known NA inhibitor zanamivir (0.8 µM) as a positive control for 30 min at 37°C. After 1-h incubation with NA-Fluor substrate at 37°C, NA activity was measured by fluorescence spectrometry. Data are presented as mean ± SD of three replicates for three independent experiments.

The effects of cotreatment (**Figure 3E**) suggest that TSN may directly affect influenza virus particle. Therefore, we examined whether TSN inhibits the HA activity of influenza A virus strains A/PR/8/34 and H3N2 required for membrane attachment. The HA inhibition assay showed that TSN did not inhibit viral attachment onto cRBCs even at 250 µM (**Figure 4A**). This result strongly suggests that TSN does not reduce infectivity by inhibiting host binding of influenza A virus. We also found that the NA activity of A/PR/8/34 and H3N2 strains was not inhibited by up to 250 µM TSN, although it was completely inhibited by the known NA inhibitor zanamivir (0.8 µM) (**Figure 4B**). Collectively, these data suggest that TSN did not inhibit the activity of HA and NA of influenza A virus, A/PR/8/34 and H3N2.

### TSN Inhibited Viral mRNA Synthesis but Did Not Affect Host-Cell Immune Response

**Figure 5:**
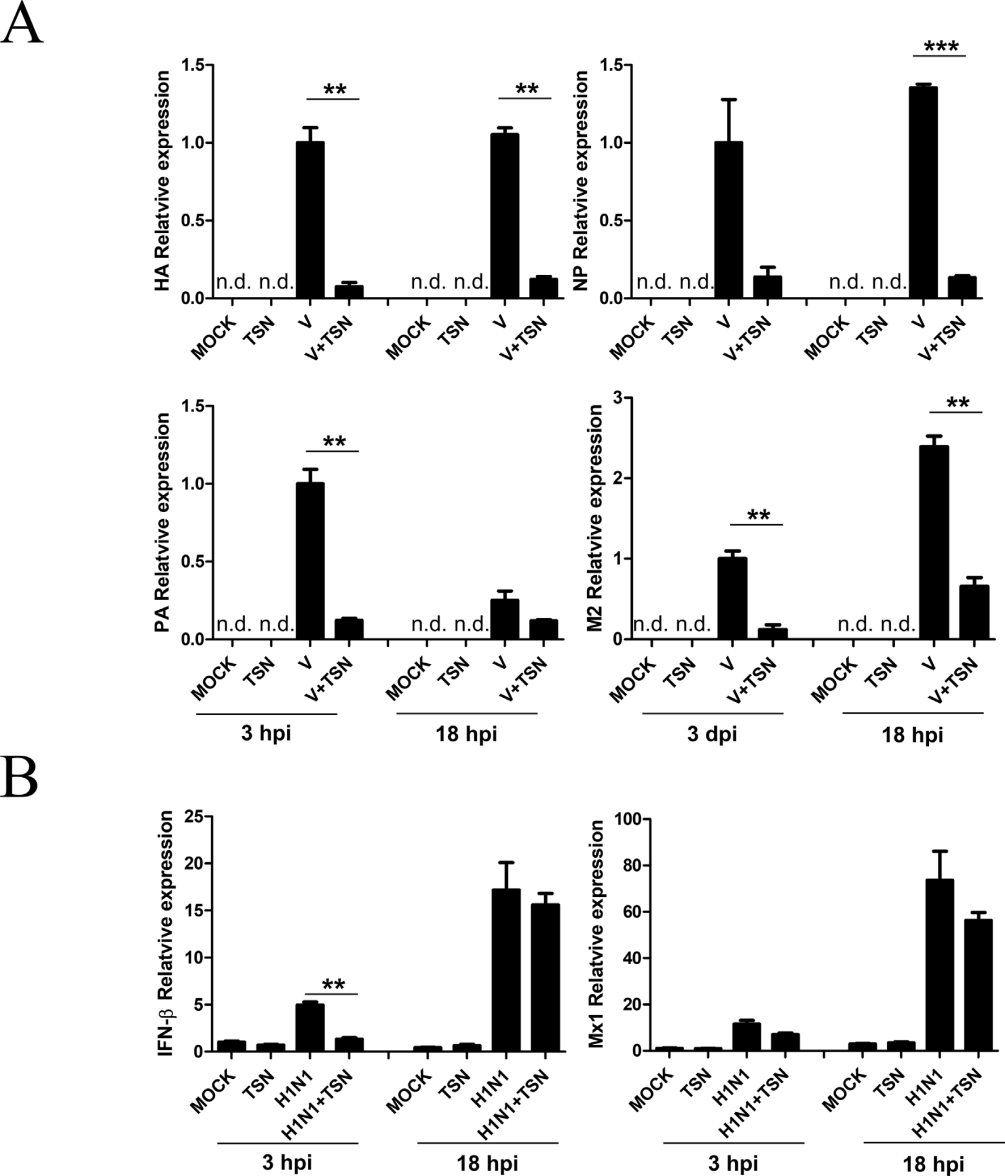
TSN pretreatment decreased synthesis of influenza virus A/PR/8/34 mRNA but did not affect the antiviral host response. **(A)** MDCK cells were pretreated with TSN (2 µM) as described then infected with 10 MOI A/PR/8/34. Expression levels of HA, NP, PA, and M2 viral mRNAs were assessed by qRT-PCR. **(B)** Host expression levels of IFN-β and Mx1 were assessed at 3 and 18 hpi by qRT-PCR. Expression levels were normalized to β-actin mRNA. Data are presented as mean ± SD of three replicates and are representative of three independent experiments (n.d., not detected). V, control virus-infected MDCK cells. Statistical significance was analyzed by Student’s *t*-test. **P < 0.01; ***P < 0.001.

We next investigated whether TSN inhibits viral mRNA synthesis. After pretreatment with 2 µM TSN, MDCK cells were infected with A/PR/8/34, harvested, and subjected to qRT-PCR at 3 and 18 hpi to assess relative mRNA expression levels of HA, NP, PA, and M2 viral genes. Levels of HA, NP, PA, and M2 viral mRNAs were decreased in TSN-pretreated MDCK cells at both times compared with virus-infected control cells (**Figure 5A**).

We also examined whether TSN treatment affects the A/PR/8/34 virus-induced host immune response by measuring the levels of IFN-β and Mx1 mRNAs in MDCK cells pretreated with 2 µM TSN at 3 and 18 hpi by qRT-PCR. Pretreatment with TSN reduced A/PR/8/34 virus-induced upregulation of IFN- β and Mx1 mRNA at 3 hpi, but IFN- β and Mx1 mRNA levels were not affected by TSN pretreatment at 18 hpi compared with virus-infected control cells (**Figure 5B**).

### TSN Decreased Viral Protein Expression and Changed PA Protein Localization

**Figure 6:**
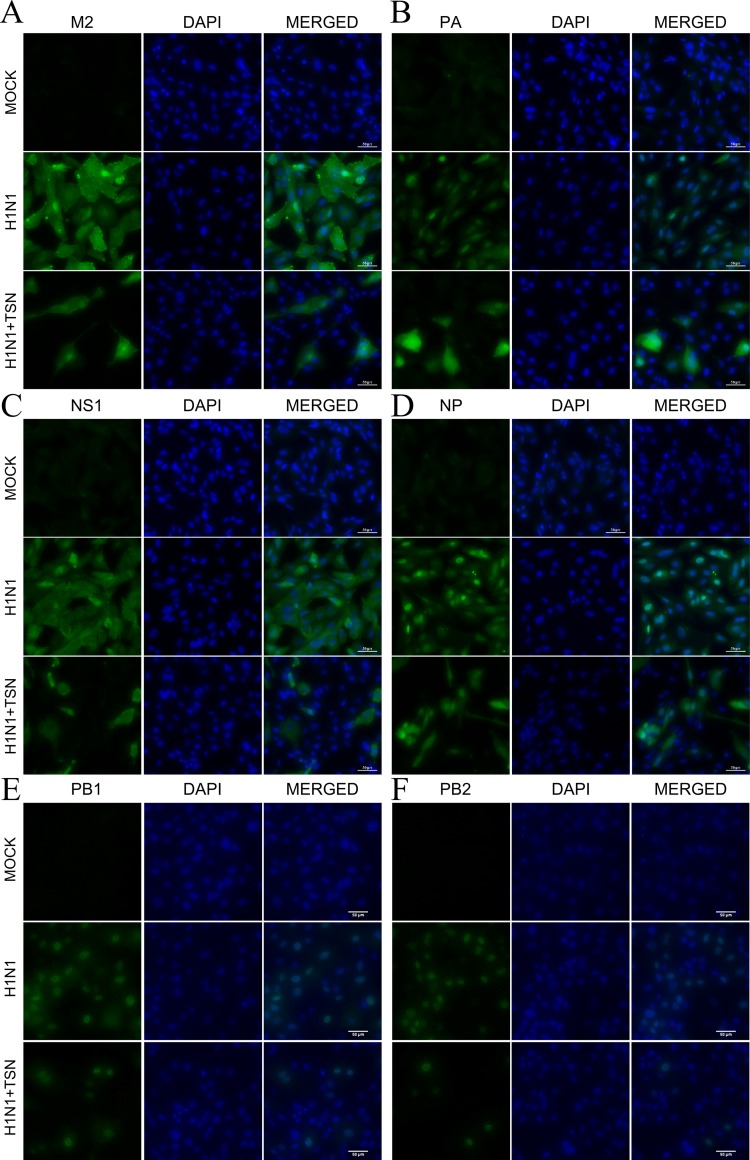
TSN pretreatment reduced viral M2, PA, NS1, NP, PB1, and PB2 protein expression levels and altered PA protein subcellular localization. MDCK cells were pretreated with TSN (2 µM) and then infected with 10 MOI A/PR/8/34. Cells were immunostained at 18 hpi. Green foci indicated the presence of M2 **(A)**, PA **(B)**, NS1 **(C)**, NP **(D)**, PB1 **(E)**, or PB2 **(F)** viral proteins (left column). The nucleus was counterstained with DAPI (blue, middle column). Merged images indicate the subcellular localizations of immunostained proteins (right column). Data are representative of three different experiments. H1N1, control virus-infected MDCK cells. Scale bars, 50 µm at ×40 magnification.

To examine whether these changes in mRNA expression also affect viral protein expression, we performed immunofluorescence staining with anti-M2, -PA, -NS1, -NP, PB1, and PB2 antibodies at 18 hpi. Consistent with qRT-PCR results, expression levels of viral M2, PA, NS1, NP, PB1, and PB2 proteins were reduced in TSN-pretreated MDCK cells at 18 hpi compared with virus-infected control cells (**Figures 6A–F**).

Viral polymerase PA proteins were localized in the nucleus of control influenza virus–infected MDCK cells as previously reported (Jones et al., 1986). However, viral polymerase PA protein, not PB1 and PB2, was localized in the nucleus and cytoplasm of TSN-pretreated influenza virus–infected MDCK cells (**Figure 6B** and **Supplementary Figure 3A**). Therefore, these data suggest that TSN pretreatment reduces viral protein expression, and PA protein was localized in nucleus and cytoplasm of the influenza A virus–infected cell.

### TSN Can Occupy the Active Site of PA Protein

**Figure 7:**
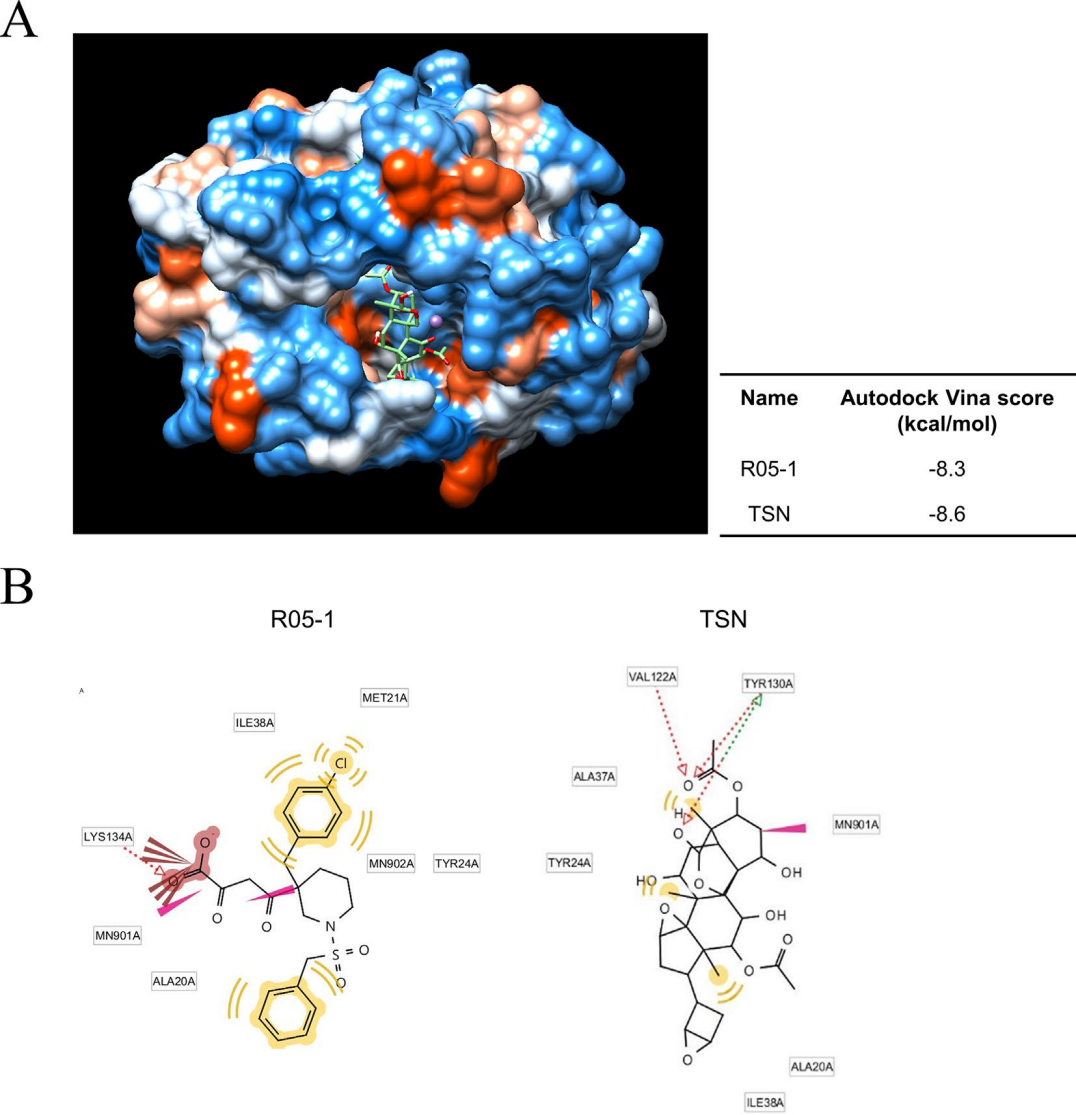
Docking and binding simulations showing that TSN can occupy the active site within the PA protein. **(A)** Computational structure prediction for docking simulation between TSN and the N-terminal domains of PA (PA-Nter) protein of influenza virus pH1N1. The small tables indicate the predicted docking score for TSN and the positive control R05-01 with the PA protein. **(B)** The binding sites of TSN and the positive control R05-01 on the PA-Nter domain are shown.

We next investigated the possible mechanisms for TSN-induced ectopic localization of PA protein. We conducted *in silico* modeling to examine if TSN can directly bind to the N-terminal domain of the pH1N1 influenza virus PA protein N-terminal domain (PA-Nter), which is critical for PA protein stability and nuclear translocation (Nieto et al., 1994; Hara et al., 2006). Docking simulations using the AutoDock Vina program predicted that the binding affinity of TSN to the PA-Nter domain is stronger than that of the known PA protein inhibitor R05-01 (binding free energy change of −8.6 *vs*. −8.3 kcal/mol for R05-01) (**Figure 7A**).

*In silico* analysis of the binding mode using LigandScout 3.0 software predicted that R05-01 may bind to the PA-Nter domain mainly by forming hydrophobic interactions with ILE38, MET21, TYR24, and ALA20, and a hydrogen bond with LYS134. Alternatively, TSN may bind primarily through hydrogen bonds with VAL122 and TYR130, and hydrophobic interactions with ALA37, TYR24, ALA20, or ILE38, possibly contributing to the higher binding affinity of TSN (**Figure 7B**). Taken together, these data suggest that TSN-mediated antiviral activity may involve PA protein inhibition by direct binding to a critical functional site (the PA-Nter domain), thereby suppressing nuclear translocation and viral transcription.

### TSN Reduced PI3K/AKT Phosphorylation Induced by Influenza Virus Infection

**Figure 8:**
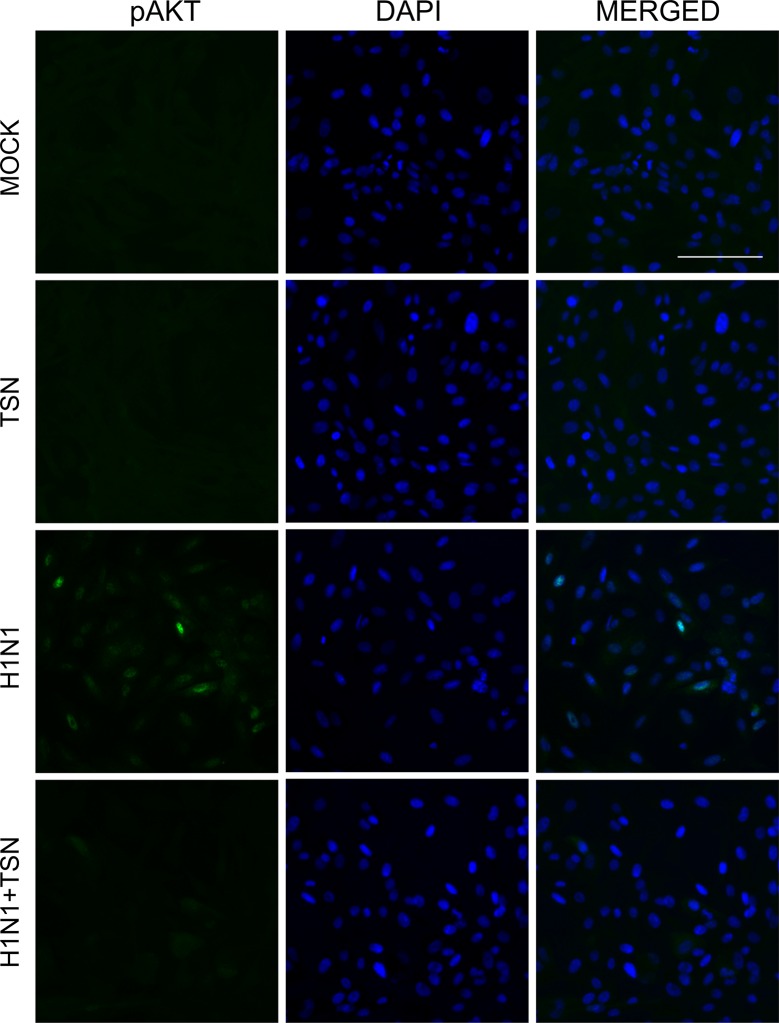
TSN pretreatment inhibited the phosphorylation of AKT induced by influenza A virus infection. MDCK cells were pretreated with TSN at 2 µM for 6 h prior to infection by 10 MOI A/PR/8/34. Cells were stained with anti-phospho-AKT antibody (left column, green) and counterstained with DAPI (middle column, blue) at 18 hpi. The phospho-AKT protein and DAPI image are merged (right column). Data are representative of three independent experiments. Scale bars, 100 µm at ×20 magnification.

It is reported that PI3K/AKT phosphorylation occurs during viral entry, and TSN suppressed the AKT/GSK-3β/β-catenin signaling pathway in colorectal cancer cells (Ehrhardt et al., 2006; Wang et al., 2015). To elucidate the effect of TNS on virus-induced PI3K/AKT signaling, we examined if TSN pretreatment alters phospho-AKT in A/PR/8/34-infected MDCK cells (**Figure 8** and **Supplementary Figure 3B**). Immunofluorescence staining revealed that influenza A virus infection induced phospho-AKT protein at 18 hpi, as previously reported (Ehrhardt et al., 2006), and that expression was reduced in TSN-pretreated virus-infected MDCK cells at 18 hpi compared with virus-infected control cells, suggesting TSN pretreatment reduced virus-induced AKT activity.

### Oral Administration of TSN Enhanced the Survival Rate of Infected Mice

**Figure 9:**
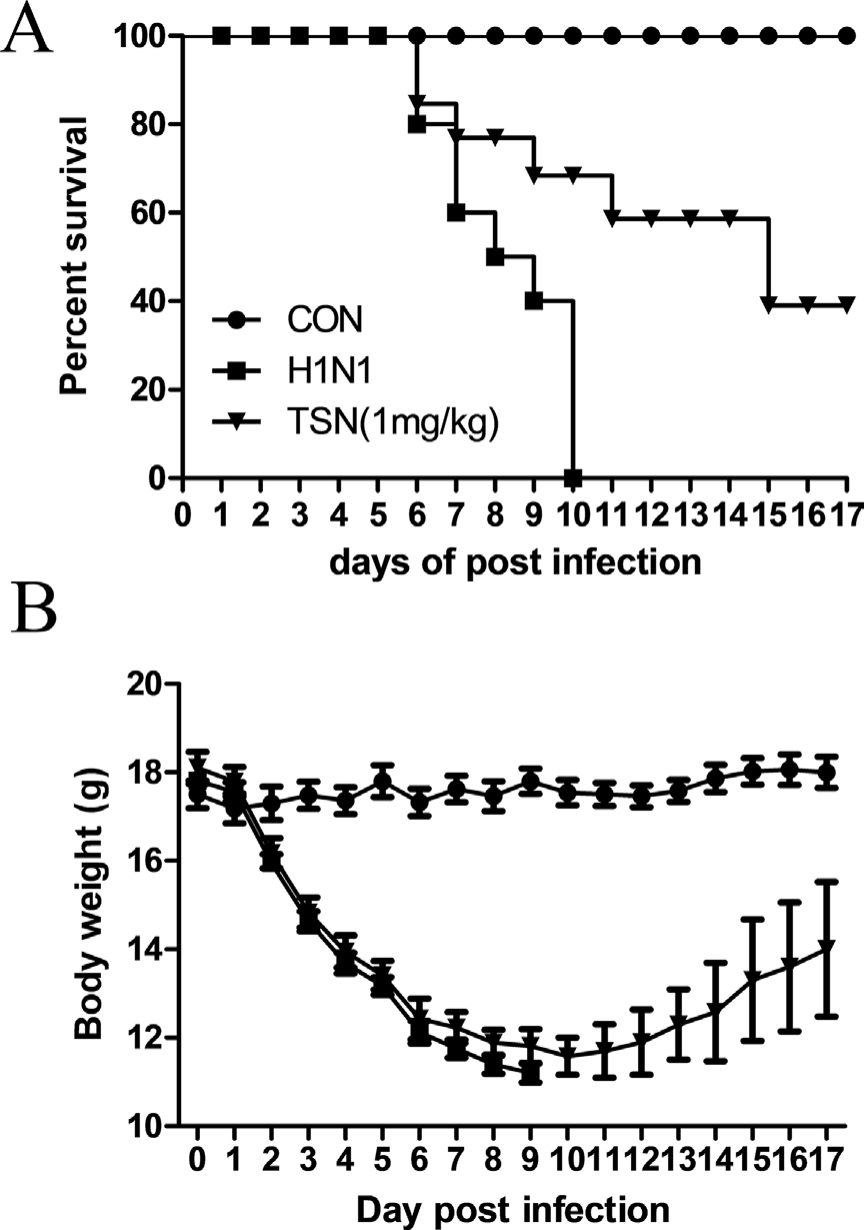
Oral administration of TSN protected Balb/c mice from influenza A virus-induced death. **(A** and **B)** TSN (1 mg/kg) was orally administered to Balb/c mice daily for 8 days before intranasal infection with mouse-adapted A/PR/8/34 at 5× LD_50_. The survival rate **(A)** and body weight **(B)** of uninfected mice (CON, *n* = 5), vehicle-treated infected mice (H1N1, *n* = 10), and TSN-pretreated infected mice (*n* = 10 at each dose, 1 mg/kg per day for 7 days) were monitored for 17 days.

Finally, we assessed the protective efficacy of TSN treatment on influenza-infected mice. Six-week-old female BALB/c mice were infected intranasally with five MLD_50_ mouse-adapted A/PR/8/34 after oral administration of vehicle or TSN and survival rate and body weight were monitored for 17 days. The majority of vehicle-treated infected mice died within 10 days postinfection (dpi) (**Figure 9A**), whereas mice pretreated daily with TSN for 8 days up to the day of infection (−7 to 0 day preinfection) exhibited 40% survival at 1 mg/kg/day (*n* = 10). Mice infected with influenza virus showed obvious weight loss (**Figure 9B**); however, mice pretreated with 1 mg/kg/day regained weight starting on 11 dpi following similar weight loss for 10 dpi.

## Discussion

Our previous study demonstrated that an ethanol extract of MF reduced influenza A virus infection, by inhibiting HA and NA activities and altering the expression or localization of multiple RNA polymerase complex subunits (Jin et al., 2017). However, the specific extract component(s) mediating this effect were not identified. In this study, we found that TSN, but not OCN, has strong antiviral activity against influenza A *in vitro* and substantially reduces the mortality of infected mice.

The marked antiviral efficacy of TSN when administered before or concurrent with virus exposure, but not when administered hours after virus exposure, suggests that TSN targets some process required during the early stage of virus infection. During the early stage of influenza A virus infection, the HA protein binds to sialyloligosaccharide on the host-cell membrane to initiate host entry (Paules and Subbarao, 2017). The NA protein enhances HA-mediated viral membrane infusion and infectivity as well as progeny release by cleaving sialyloligosaccharide (Su et al., 2009). However, TSN did not inhibit HA or NA protein activity (**Figure 4**). It was reported that the PI3K-AKT signaling pathway regulates the very early steps of viral entry and that AKT inhibition suppresses viral entry and replication (Ehrhardt et al., 2006; Hirata et al., 2014), suggesting another possible target for TSN. Indeed, TSN suppressed the AKT/GSK-3β/β-catenin signaling pathway in colorectal cancer cells, resulting in growth inhibition and apoptosis (Wang et al., 2015). We also found that phosphorylation of AKT induced by influenza virus infection was reduced by TSN pretreatment, suggesting that TSN interferes with the early stage of viral entry by inhibiting AKT activity (**Figure 8**).

In addition to reducing AKT signaling, TSN pretreatment inhibited viral HA, NP, PA, and M2 mRNA synthesis at 3 and 18 hpi and reduced expression of viral proteins NP, PA, M2, and NS1 (**Figures 5** and **6**). Moreover, the subcellular localization of PA protein was altered by TSN pretreatment. PA protein was reported to localize in the nucleus of influenza virus–infected MDCK cell (Jones et al., 1986), but TSN enhanced cytoplasmic localization, suggesting disruption of translocation. The N-terminal domain of PA protein (PA-Nter) (amino acid 1 to 209) is critical for protein stability and endonuclease activity, and it has also been reported that region I (amino acids 124 to 139) and region II (amino acids 186 to 247) of PA protein are involved in nuclear translocation (Nieto et al., 1994; Hara et al., 2006). Our *in silico* docking analysis and mode of action study suggested that the binding affinity of TSN may be stronger than that of the known PA protein inhibitor R05-01 and that TSN may bind to the PA-Nter domain primarily through hydrogen bonds with VAL122 and TYR130, the residues in the vicinity of nuclear translocation signal region I (amino acids 124 to 139) (**Figure 7**). Therefore, binding of TSN to PA protein may block accessibility of the nuclear translocation signal, resulting in cytoplasmic accumulation of PA protein and inhibition of influenza virus mRNA replication. These multilevel antiviral effects of TSN were supported by the increased survival rate of influenza-infected mice receiving oral TSN pretreatment (**Figure 9**).

In contrast to TSN, our previous study showed that MF treatment inhibited influenza A virus infection by HA and NA protein activity inhibitions, the accumulation of PA, PB1, and PB2 protein and increased expression of Mx1 antiviral protein (Jin et al., 2017). This inconsistency suggests that MF contains multiple antiviral compounds with distinct modes of action and that the mode of MF action depends on the specific concentrations of these compounds. Further study is required to identify other antiviral compounds in MF ethanol extract, their modes of action, and dose dependency. In summary, this study suggests that TSN from MF can inhibit influenza virus infection at an early stage by blocking PA protein nuclear translocation. Given the efficacy against influenza A virus *in vitro* and *in vivo*, TSN could be a promising candidate PA-targeting anti-influenza drug.

## Data Availability

All datasets generated for this study are included in the manuscript/**Supplementary files**.

## Ethics Statement

The animal study was reviewed and approved by All animal protocols were approved by the Institutional Animal Care and Use Committee of the Daegu-Gyeongbuk Medical Innovation Foundation (DGMIF), Daegu, South Korea (approval number, DGMIF-17031401-01).

## Author Contributions

Y-HJ designed experiments, performed experiments and wrote manuscript. SK and J-GC performed experiments. BL performed *in silico* analysis. JM and W-KC proofread manuscript.

## Funding

This research was supported by Basic Science Research Program through the National Research Foundation of Korea (grant no. NRF-2017R1D1A1B03032119), the Korea Institute of Oriental Medicine (grant no. K17281, G18201 and G18202) and the National Research Council of Science & Technology (NST) grant (No. CRC-16-01-KRICT) funded by the Korea government (MSIT).

## Conflict of Interest Statement

The authors declare that the research was conducted in the absence of any commercial or financial relationships that could be construed as a potential conflict of interest.
